# Immunoproteomic profile of *Trichinella spiralis* adult worm proteins recognized by early infection sera

**DOI:** 10.1186/s13071-015-0641-8

**Published:** 2015-01-13

**Authors:** Jing Yang, Wei Pan, Ximeng Sun, Xi Zhao, Gu Yuan, Qing Sun, Jingjing Huang, Xinping Zhu

**Affiliations:** Department of Parasitology, School of Basic Medical Sciences, Capital Medical University, Beijing, China

**Keywords:** *Trichinella spiralis*, Adult worm, Mass spectrometry, Immunoproteomics, Diagnosis

## Abstract

**Background:**

Trichinellosis, a widespread zoonosis, is regarded as an emerging or reemerging disease. Effective treatment and prognosis of trichinellosis depends on early diagnosis of the infection. The objective of this study was to identify sensitive and specific antigens for early diagnosis or effective vaccine antigens for preventing infection.

**Methods:**

The somatic proteins of *T. spiralis* adult worms were separated by two-dimensional gel electrophoresis (2-DE). The separated proteins were probed with early infection sera from swine or mice infected with *T. spiralis* for 7 days. The primary immunoreactive spots were characterized by MALDI-TOF/TOF-MS analysis in combination with bioinformatics. The identified proteins were annotated using WEGO based on their functions. The immunodominant protein was chosen for expression as recombinant protein in *E. coli* and the purified recombinant protein was used to confirm its antigenicity by Western blot with the same infection sera.

**Results:**

Approximately 300 spots were separated by 2-DE, with molecular weights ranging from 10 to 130 kDa, and pI values ranging from pH 4 to 10. The sera from swine and mice infected with *T. spiralis* for 7 days recognized 64 proteins. MALDI-TOF/TOF-MS analysis identified 55 proteins, some with different isoforms. Finally, 40 individual immunoreactive proteins were obtained with a wide range of biological functions. Several proteins, such as heat shock protein 70, 14-3-3 protein, and cysteine protease could be used as immunodiagnostic or vaccine antigens. Among these identified proteins, the highly immunodominant *Ts*14-3-3 was chosen for expression in *E. coli* and purified recombinant *Ts*14-3-3 was able to be strongly recognized by the same *T. spiralis* infected sera used for identifying these antigens, therefore the most promising antigen for early immunodiagnosis of *Trichinella* infection.

**Conclusions:**

A total of 64 proteins from the adult worm were recognized by early infection sera from swine and mice infected with *T. spiralis* for 7 days. Several proteins, are of particular interest as immunodiagnostic or vaccine antigens, especially with *Ts*14-3-3 as most promising due to its highly immunogenicity during early infection, expressed protein can be recognized by *Trichinella* early infection sera and the native *Ts*14-3-3 expression in both adult and larval stages.

## Background

Trichinellosis, which is caused by infection with the nematode *Trichinella spiralis* remains an important food-borne parasitic zoonosis worldwide. It not only causes a public health hazard but also represents an economic problem in porcine animal production and food safety [1,2]. Human trichinellosis is primarily caused by ingesting the raw or undercooked meat of pigs and other animals containing *T. spiralis* larvae [3]. The disease affects as many as 11 million people and is regarded as a re-emerging disease [4]. From 2005 to 2009 in China, 15 outbreaks of human trichinellosis were reported, with 1387 cases and four deaths [5]. *T. spiralis* is a tissue-dwelling parasitic nematode, and its life cycle is completed in a single host, including all stages of the adult worm (Ad), newborn larvae (NBL) and muscle larvae (ML). After ingestion, muscle larvae are released from the capsules and develop into sexually mature adult worms in the intestine. Each female worm can produce 500-1000 NBL in a period of 5-10 days. Then, the NBL migrate through the lymphatic system and the general circulatory system. Subsequently, the parasites develop into infective muscle larvae encapsulated in the host skeletal muscles [6]. During all developmental stages, *T. spiralis* expresses many immunodominant antigens that elicit a protective immune response, as well as antigens useful for the serodiagnosis of trichinellosis [7,8].

Because trichinellosis manifests as nonspecific signs and symptoms, the clinical diagnosis of this disease is difficult [9]. Currently, the diagnosis of trichinellosis relies on detection of the larvae in a muscle biopsy or highly specific immunodiagnostic tests. The immunological tests current available use excretory-secretory products (ES) secreted by the living larvae as antigens to detect antibodies in the sera of infected individuals. However, testing is not widely available because of the limited availability of antigens produced by *T. spiralis* larvae. These tests are also not sensitive for the detection of the early stage of infection and may cross-react with other parasites [10,11]. Another weakness of serologic methods using ES products as antigens is the high rate of false negative results, even with a combination of ELISA and Western blot, which may increase the detection sensitivity [10,11]. Anti-helminthic drugs are more effective against the adult worms remaining in the intestine during early infection than the encapsulated muscle larvae. After the newborn larvae are released by the adult worm, the NBL migrate to the muscle tissue to be encapsulated that are more resistant to anthelmintic therapy and cause greater pathology; therefore, it is critical to identify and diagnose early infection for timely treatment [9,12]. The antigens from the early stage of infection, such as adult worms and newborn larvae, are immunogenic and induce immune responses in an infected host and may be used for early immunodiagnosis [7]. Although previous studies showed that some antigens derived from newborn larvae are recognized by *T. spiralis*-infected animal sera or have potential as vaccine antigens [13,14], these antigens have not shown satisfactory results for detecting early infections or vaccine development. Therefore, it is necessary to identify sensitive and specific novel antigens derived from adult worms or newborn larvae to detect early infection by *T. spiralis* to achieve better therapeutic effects.

Two-dimensional electrophoresis (2-DE), combined with mass spectrometry (MS) is an effective approach for high resolution analysis of complex groups of proteins [15]. Combining these techniques with Western blot using a specific antibody allows us to identify antigens that induce host immune responses during infection and could therefore be used for immunodiagnosis and/or vaccine development. This immunoproteomics tool provides information on both the characteristics of immunogenic proteins and the serological response directed against parasites, such as *Schistosoma japonicum* [16], *Toxoplamsma gondii* [17], *Ascaris lumbricoides* [18] and *Taenia solium* [19]. In fact, several excretory-secretory (ES) proteins and surface proteins from *T. spiralis* muscle larvae were identified by using this immunoproteomic tool [20-22]. These results suggest that proteins, such as serine protease, p43 glycoprotein and 5′-nucleotidase, are potential diagnostic antigens and the targets for vaccines in host-parasite interactions. However, these antigens are derived from muscle larvae and may not be suitable for the early diagnosis of *Trichinella* infection.

In this study, to identify antigens recognized by the host immune system during early enteric infection, *T. spiralis* adult worm extracts were separated by 2-DE and recognized using sera from pigs and mice infected with *T. spiralis* for 7 days. The primary immunoreactive spots recognized using the early infection sera were characterized by MALDI-TOF/TOF-MS analyses in combination with bioinformatics. The antigens recognized by the sera during this early stage may be used to immunodiagnose the early infection by *T. spiralis*. The results of this study will facilitate the selection of antigens as reagents for early serological diagnosis and possibly vaccine development.

## Methods

### Animals

Experimental animals (mice and pigs) were purchased from the Laboratory Animal Services Center of Capital Medical University (Beijing, China). Experimental procedures for *T. spiralis* infection were reviewed and approved by the Capital Medical University Animal Care and Use Committee and were consistent with the NIH Guide for the Care and Use of Laboratory Animals.

### Maintenance and collection of *T. spiralis* worms and protein sample preparation

The *T. spiralis* ISS 533 strain used in this study was maintained in female ICR mice in our laboratory, and *T. spiralis* muscle larvae were recovered from the muscles of infected mice with a standard pepsin/hydrochloric acid digestion method [23]. The adult worms were collected from the small intestine of experimentally infected Wistar rats at 4 days after experimental infection.

The collected adult worms were washed several times in PBS and then homogenized in lysis buffer containing 7 M urea, 2 M thiourea, 4% CHAPS, 65 mM Tris, 2% DTT, and 1% Bio-Lyte (pH3-10). The adult worm lysates were centrifuged at 20,000 × g at 4°C for 60 min and the supernatant was collected as adult extracts. The total protein concentration was determined using a 2D Quant kit (GE Healthcare, USA) and stored at -80°C until use for proteomic analysis.

### Collection and preparation of *T. spiralis* infected animal sera

*T. spiralis* infected pig sera were collected from 4 pigs orally infected with 20,000 *T. spiralis* muscle larvae each for 7 days and pooled. The infected mouse sera were obtained from 10 BALB/c mice orally infected each with 500 *T. spiralis* muscle larvae for 7 days. Uninfected sera from normal pigs and mice were collected as negative controls.

### Two-dimensional polyacrylamide gel electrophoresis (2-DE)

Approximately, 400 μg of *T. spiralis* adult extracts were used for each 2-DE analysis. The worm extracts with bromophenol blue were loaded into 17 cm pH 3-10 immobilized pH gradient (IPG) strips (Bio-Rad, USA) and separated by isoelectric focusing (IEF) using a Protean IEF Cell (Bio-Rad, USA). IEF was performed at 20°C with rehydration at 20°C for 12 h, followed by isoelectric focusing according to the manufacturer’s instructions. After isoelectric focusing, the IEF strips were equilibrated for 15 min in a reducing buffer (6 M urea, 2% SDS, 0.375 M Tris-HCl pH 8.8, 20% glycerol, and 2% DTT), followed by 15 min in an alkylation buffer (6 M urea, 2% SDS, 0.375 M Tris-HCl, pH8.8, 20% glycerol, and 2.5% iodoacetamide). For the second dimension, the equilibrated IPG strips were placed on 12% SDS-PAGE and run at 15 mA/gel for 30 min. The gels were either stained with Coomassie Blue R-350 or transferred to a PVDF membrane for Western blot. Three gels were run for the adult worm extracts under the same conditions.

### Western blot analysis

The separated protein spots from the 2-DE gels were transformed onto PVDF membranes (Millipore, USA) using a Trans-blot semi-dry Transfer Cell TM (Bio-RAD, USA) for 90 min at 18 V. The membranes were blocked with 5% skimmed milk in PBS, pH 7.4 for 2 h at room temperature and then incubated with either *T. spiralis* infected swine pooled sera or infected mouse sera diluted 1:1 00 in PBS, pH7.4, with 0.05% Tween 20 (PBST). After 3 washes in PBST, the membranes were incubated with the horseradish peroxidase-conjugated rabbit anti-swine IgG or goat anti-mouse IgG (Sigma, USA). Reproducibility of the immune recognition was verified by repeating the immunoblot at least three times. The sera collected from normal swine or mice were used as controls. The PDQuest™ 2-D Analysis Software (Bio-RAD, USA) was used for matching and analysis of the antigenic protein spots on the 2-D gels.

### In-gel tryptic digestion

Only those spots recognized by the infection sera from both the pigs and mice were selected for further study. Following the selection of spots of interest by matching with the immunoblot image, the protein spots on the Coomassie Blue-stained gels were carefully excised. The excised gel pieces were incubated with 100 mM NH_4_HCO_3_ and 30% CAN in the dark, washed with 100 mM NH_4_HCO_3_ and dried. Then, the gel species were further rehydrated in digestion buffer containing trypsin (Promega, USA) and incubated at 37°C overnight. The trypsin-digested peptides were subsequently extracted with 100 μl of 60% ACN/0.1% trifluoroacrtic acid (TFA) and prepared for MALDI-TOF/TOF-MS analysis.

### Protein identification using MALDI-TOF /TOF-MS analysis

The resulting peptides from the above extraction were analyzed on an Applied Biosystem Sciex 4800 mass spectrometer (Applied Biosystems, USA). The MS spectra were recorded in reflector mode in a mass range from 800 to 4000. The peptide mass fingerprints (PMFS) were obtained. The peaks with the highest mass intensities were selected for MS/MS for protein identification. Each peptide assignment was also manually verified. The PMFS and MS/MS data were searched from the NCBI and SwissProt databases of the existing *T. spiralis* genome or ESTs using MASCOT version 2.2 (Matrix Science, LTD). Protein identification was considered accurate when the confidence scores >95% and the MASCOT score was >50. The identified proteins were categorized by their protein sequences according to the information obtained from the InterProsan software. The outputs were subjected to analysis by the GO categories using the Web Gene Ontology Annotation Plotting (WEGO, http://wego.genomics.org.cn/cgi-bin/wego/index.pl).

### Expression and immunological test of recombinant *Ts*14-3-3 (r*Ts*14-3-3)

DNA coding for the full length *Ts*14-3-3 was amplified from total cDNA reverse-transcribed from total *T. spiralis* adult RNA using primers designed according to *Ts*14-3-3 sequence (GenBank accession no. XM_003378886) (the forward primer: 5′-CGGGATCCATGACCGAAAAGGAAGACAT-3′ and the reverse primer: 5′-CGGAATTCCTGCCCAGCGGCTGTATCTT-3′). The amplified DNA products were cloned into the expression vector pET-28a (+) (Novagen, Germany) using BamHI and XhoI sites. The recombinant pET-28a (+)/*Ts*14-3-3 plasmids were transformed into the *E. coli* BL21. The recombinant protein (r*Ts*14-3-3) was induced with 1 mM IPTG and the expressed soluble recombinant *Ts*14-3-3 was purified using His-Bind Purification Kit (Novagen, USA).

Following SDS-PAGE, the r*Ts*14-3-3 was transferred onto nitrocellulose membrane. The membrane was incubated with the same *T. spiralis*-infected swine or mice sera used for identifying the immunogenic antigens described above. The recombinant *Ts*14-3-3 was used to immunize mouse to obtain antiserum in order to detect the expression of native *Ts*14-3-3 in adult worm and muscle larvae stages of *T. spiralis*.

## Results

### 2-DE analysis of *T. spiralis* adult extracts

**Figure 1:**
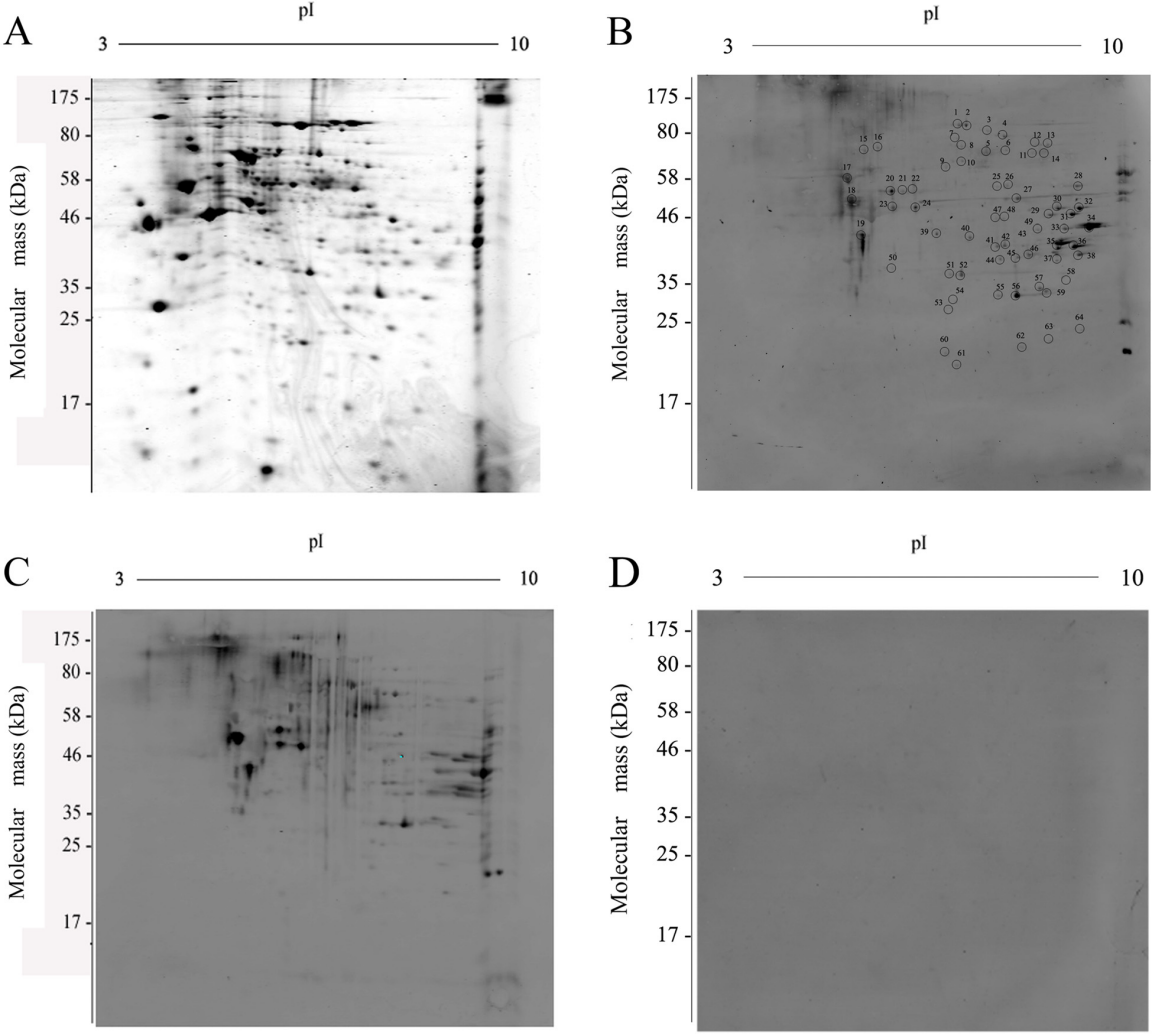
**2-DE and Western blot analysis of** ***T. spiralis*** **adult worm proteins. A**. 2-DE gel of adult worm proteins separated in the first dimension in the pH range 3-10 and in second dimension on a 12% polyacrylamide gel. The 2-DE gel was stained with Coomassie Blue G-350. **B**. Western blot of adult worm proteins probed with pig infection sera at 7 days post infection. The protein spots selected for further analysis are numbered. **C**. Western blot of adult worm proteins probed with mouse infection sera at 7 days post infection. **D**. Western blot of adult worm proteins probed with normal swine sera.

The somatic proteins of the *T. spiralis* adult extracts were separated on a 2-DE gel, and the protein spots were visualized following Coomassie R-350 staining (Figure 1A). Image analysis revealed more than 300 spots stained on the gel, the majority of which migrated between pH 4 and 10 over a broad range of molecular masses between 10-150 kDa.

### Immunoreactive antigens recognized by early infected swine and mouse sera

The PDQuest software allowed us to overlap shared spots between the 2 species, swine and mouse, based on the same MWs and isoelectric points. As shown in Figure 1B and Figure 1C, there were more than sixty protein spots recognized by sera from both swine and mice infected with *T. spiralis* for 7 days, with molecular weights ranging from 20 kDa to 180 kDa, and pI values from 3-10. Most of the recognized antigens were between 30 kDa to 150 kDa, with pI values between 4 and 9. The spots and their corresponding proteins on the gel were labeled individually with the same number. No proteins reacted to normal swine or mouse serum (Figure 1D).

### Identification of protein spots by MALDI-TOF MS analysis

The immunoblot and the Coomassie Blue stained gel were aligned and matched with the PDQuest™ 2-D Analysis Software. Sixty-four of the positive spots recognized by swine and mice sera infected with *T. spiralis* for 7 days were matched and located on the Coomassie blue stained gel, and then excised for in-gel tryptic digestion. The digested peptides were then subjected to MALDI-TOF/TOF-MS analysis. The proteins listed in the table are the highest ranked candidates that were unambiguously identified in the MASCOT search (Table 1) with molecular weights listed from high to low. A total of 55 immunoreactive proteins were identified by peptide mass fingerprinting. Nine spots selected for MS analysis did not generate any MS data. Several proteins, such as the intermediate filament protein, actin-5c, 14-3-3, inorganic pyrophosphatase, putative thioredoxin, and serine protease inhibitor were identified in several spots next to each other. Actin-5c was identified in spots 19, 20, 21, and 22. The 14-3-3 protein was found in spots 60 and 61. Putative thioredoxin was identified in spots 42, 51 and 52. Spots 56, 57 and 59 were the serine protease inhibitor kazal-type 4. As a consequence, a total of 40 distinct immunoreactive proteins were identified. Notably, several house-keeping genes were identified, such as actin and intermediate filament protein. Several proteins were involved in bioprocessing, metabolism and signaling pathways, such as cysteine proteases, inorganic pyrophosphatase protein-tyrosine phosphatase, heat shock protein and developmental-regulated GTP-binding protein.

**Table 1:** **Identification of** ***T. spiralis*** **adult worm proteins recognized by pig and mouse infection sera at 7 DPI**

**Spot no.**	**Protein name**	**NCBI ID**	**pI**	**MW (kDa)**	**Protein score**	**Protein score C.I.%**	**Coverage (%)**	**No. matched peptides**
1	intermediate filament protein ifa-1	gi|316973417	5.96	73.218	816	100	62	34
2	6-phosphofructokinase	gi|316973417	7.97	93.66	93	100	15	11
3	heat shock protein 70 (*Trichinella britovi*)	gi|2104672	5.77	71.86	33	99.5	18	11
4	MAP/microtubule affinity-regulating kinase 3	gi|316978564	9.25	78.23	115	100	18	11
5	chaperone protein DnaK	gi|316972817	5.43	73.47	494	100	36	22
6	32 kDa beta-galactoside-binding lectin (Galectin-1)	gi|316970057	9.04	55	85	100	17	7
7	F-box/WD repeat-containing protein sel-10	gi|316975364	6.29	67.25	67	100	19	9
8	HAD-superfamily hydrolase	gi|316976388	7.8	66.01	56	98.7	10	6
9	cysteine protease ATG4B	gi|316968242	5.79	47.54	68	100	20	6
10	developmentally-regulated GTP-binding protein 1	gi|316965924	8.97	48.51	52	99.6	20	7
11	dolichyl-diphosphooligosaccharide—protein glycosyltransferase subunitSTT3A	gi|316976331	8.85	62.448	62	97.996	25	11
12	HAD-superfamily hydrolase	gi|316976388	7.8	66.01	56	98.7	10	6
13	putative glutaredoxin-like protein	gi|316970937	6.21	91.79				13
14	MYND finger protein	gi|316977375	8.8	56.83	64	100	12	5
15	intermediate filament protein ifa-1	gi|316973417	5.96	73.21	478	99.9	51	31
16	acyl-protein thioesterase	gi|316970759	5.02	55.8	108	100	13	5
17	putative stress-induced-phosphoprotein 1	gi|316977783	6.01	58.16	93	100	22	9
18	7 transmembrane receptor	gi|316965614	5.82	41.87	89	100	38	14
19	Actin-5c	gi|316970244	5.3	42.21	280	100	36	10
20	Actin-5c	gi|316970244	5.3	42.21	422	100	34	9
21	Actin-5c	gi|316970244	5.3	42.21	133	100	17	5
22	Actin-5c	gi|316970244	5.3	42.21	133	100	17	5
23	inorganic pyrophosphatase	gi|316967781	5.76	41.796	96	100	12	4
24	inorganic pyrophosphatase	gi|316967781	5.76	41.796	96	100	12	4
25	No identified							
26	No identified							
27	adenosine deaminase	gi|47934208	5.93	39.787	61	97.4	13	4
28	putative LIM and SH3 domain protein	gi|316973491	9.47	33.96	63	98.7	33	9
29	hypothetical protein Tsp_11860	gi|316969203	8.09	39.25	65	98.2	25	8
30	hypothetical protein Tsp_11860	gi|316969203	8.09	39.25	68	97.8	25	8
31	ubiquitin-conjugating enzyme E2 1	gi|316968002	7.07	41.31	65	100	19	8
32	phosphomannomutase 2	gi|316975006	5.76	31.86	379	100	43	12
33	zinc metallo protein	gi|316965635	5.93	32.23	73	100	23	6
34	hypothetical protein Tsp_11358	gi|316966928	9.51	34.91	82	100	34	8
35	Tas retrotransposon peptidase A16 superfamily protein	gi|316959639	8.6	37.73	64	99.8	23	9
36	putative trypsin	gi|316978020	8.53	39.6	89	100	23	7
37	No identified							
38	protein-tyrosine phosphatase	gi|316967260	5.92	25.75	94	100	21	5
39	putative nudix hydrolase 6	gi|339244291	5.21	32.98	63	98.44	15	4
40	Actin-depolymerizing factor 2	gi|316972221	6.37	40.828	123	100	10	4
41	26S protease regulatory subunit 6B	gi|316968151	5.47	39	88	100	19	5
42	putative thioredoxin	gi|316967722	5.56	36.88	217	100	34	8
43	26S protease regulatory subunit 7	gi|316970971	5.67	49	97	100	22	9
44	vacuolar proton pump subunit E	gi|316974496	5.8	26.14	163	100	30	5
45	inositol monophosphatase	gi|316978221	5.65	23.35	185	100	38	5
46	No identified							
47	hypothetical protein Tsp_11860	gi|316969203	8.09	39.25			25	8
48	hypothetical protein Tsp_11860	gi|316969203	8.09	39.25			25	8
49	No identified							
50	No identified							
51	putative thioredoxin	gi|316967722	5.56	36.88	217	100	34	8
52	putative thioredoxin	gi|316967722	5.56	36.88	217	100	34	8
53	No identified							
54	tumor protein D52	gi|316971259	5.98	16.889	84	97.9	59	7
55	No identified							
56	serine protease inhibitor Kazal-type 4	gi|316969591	8.56	24.89	72	99.875	27	4
57	serine protease inhibitor Kazal-type 4	gi|316969591	8.56	24.89	72	99.875	27	4
58	No identified							
59	serine protease inhibitor Kazal-type 4	gi|316969591	8.56	24.89	72	99.875	27	4
60	14-3-3 protein	gi|257219670	4.83	28.29	74	99.855	24	6
61	14-3-3 protein	gi|257219670	4.83	28.29	74	99.855	24	6
62	tumor protein D52	gi|316971259	5.98	16.889	84	97.9	59	7
63	translation initiation factor eIF-5A	gi|316973471	5.17	12.32	113	100	57	4
64	tumor protein D52	gi|316971259	5.98	16.889	84	97.9	59	7

### Distribution of the identified protein spots

**Figure 2:**
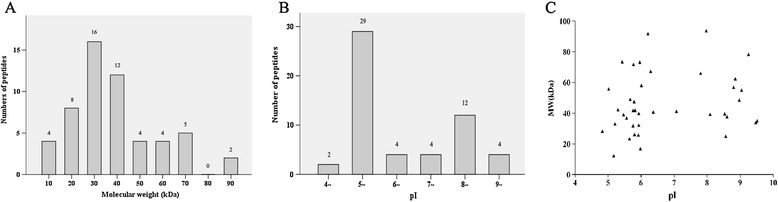
**Distributions of** ***T. spiralis*** **adult worm proteins spots recognized by both pig and mouse anti-** ***T. spiralis*** **sera at 7 DPI. (A)** Distributions of molecular weight (MW) for *T. spiralis* adult worm proteins recognized by both pig and mouse anti-*T. spiralis* sera at 7 DPI. **(B)** Distributions of isoelectric point (pI) for *T. spiralis* adult worm protein spots recognized by both pig and mouse anti-*T. spiralis* sera at 7 DPI. **(C)** The two-dimensional distribution of *T. spiralis* adult worm protein spots recognized by both pig and mouse anti-*T. spiralis* sera at 7 DPI.

The molecular weight (MW) and isoelectric point (pI) of the identified proteins were analyzed (Figure 2A). The MW of 55 proteins was ranged from 12.32 to 93.66 kDa. Twenty-eight of 55 (50.9%) proteins ranged from 30 to 50 kDa. The pI was between 4.83 and 9.04, and 29 of 55 (52.7%) proteins were in the range of pI 5 ~ 6 (Figure 2B). The two-dimensional distribution of the 55 identified proteins is shown in Figure 2C.

### Gene ontology analysis

**Figure 3:**
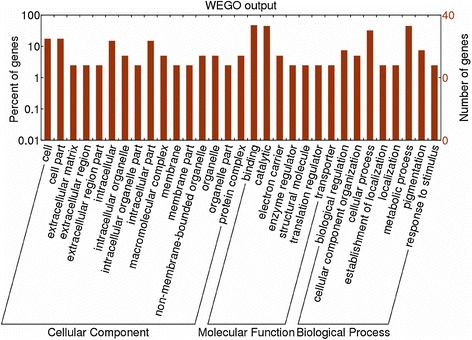
**GO categories of** ***T. spiralis*** **immunoreactive adult worm proteins.** The proteins were classified into cellular component, molecular function and biological process by WEGO according to their GO signatures.

To further understand the functions of these identified immunoreactive proteins, the InterPro database was searched based on the BLASTP results. A total of 40 proteins, not including the 15 repeated protein spots, were categorized by cellular component, molecular function, and biological process using WEGO. The primary molecular functions were catalytic activity (GO: 0003824) and binding (GO: 0005488). These proteins were classified into eight categories related to their biological process as follows: biological regulation, cellular component organization, cellular process, establishment localization, metabolic process, pigmentation, and response to stimulus. For the biological process function, a large proportion was metabolic process (GO: 0008152) and cellular process (GO: 0009987). These results are shown in Figure 3.

### Expression feasibility of r*Ts*14-3-3 and its recognition by infected sera

**Figure 4:**
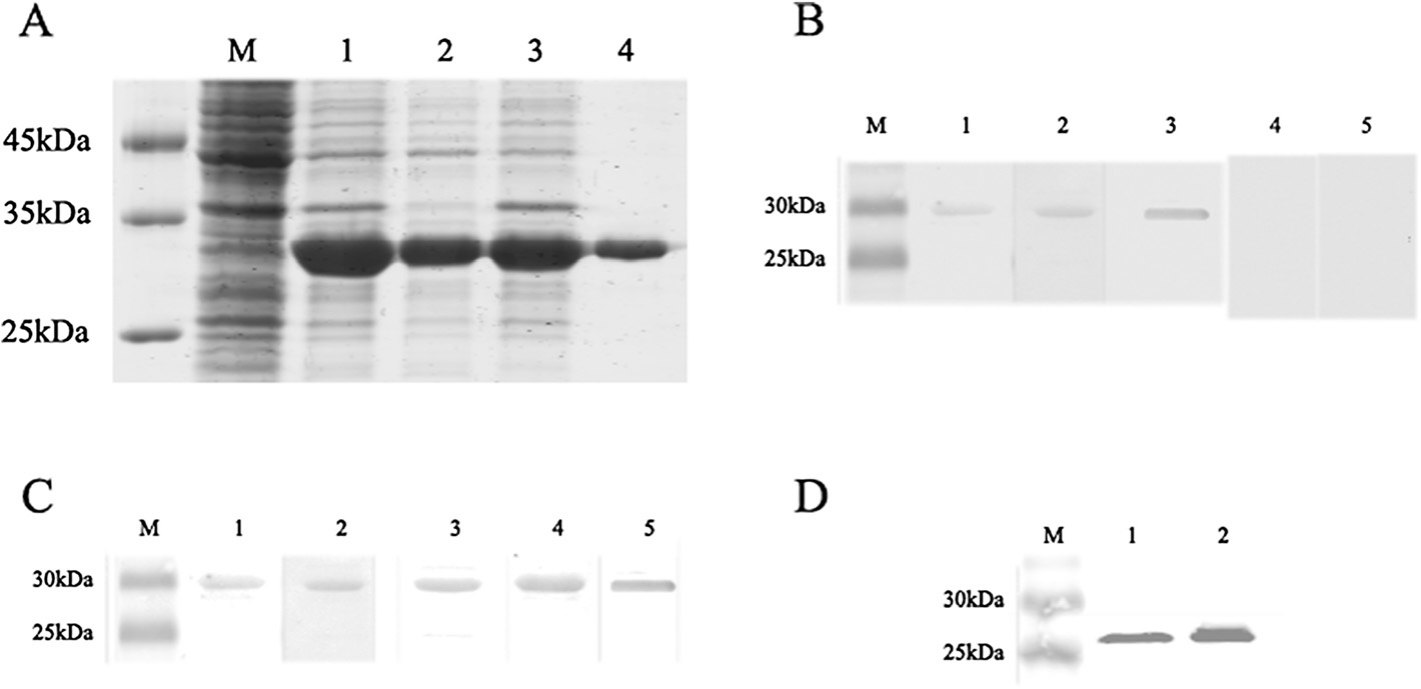
**SDS-PAGE and Western blot analysis of r** ***Ts*** **14-3-3 expressed in** ***E. coli*** **BL21. (A)** Bacterial lysates or purified recombinant protein were analyzed by SDS–PAGE and stained with Coomassie blue. Lane M, protein marker; lane 1, uninduced *E. coli* lysate; lane 2, IPTG-induced *E. coli* lysate; lane 3, insoluble fraction of induced *E. coli* lysate ; lane 4, soluble fraction of induced *E. coli* lysate ; lane 5, r*Ts*14-3-3 purified by Ni-affinity chromatography. **(B)** Western blot analysis of r*Ts*14-3-3. The r*Ts*14-3-3 was transferred on individual lane and recognized by *T. spiralis* infected swine sera at 7 dpi (lane 1); *T. spiralis* infected mouse sera at 7 dpi (lane 2) and anti-His monoclonal antibody (lane 4). **(C)**. The r*Ts*14-3-3 was recognized by sera of swine infected with *T. spiralis* for 7 days (lane 1); 14 days (lane 2); 21 days (lane 3) and 28 days ( lane 4) or by anti-His antibody as control (lane 5). **D**. Western blot analysis of native *Ts*14-3-3 in adult extracts (lane 1) and in muscle larval extracts (lane 2) of *T. spiralis* by antibody against r*Ts*-14-3-3.

To validate the potential of *Ts*14-3-3 as vaccine or diagnostic antigen, the *Ts*14-3-3 was successfully cloned and expressed in *E. coli* BL21 as soluble recombinant protein. As shown in Figure 4A, the recombinant protein (r*Ts*14-3-3) was highly induced in BL21 in soluble and insoluble fractions The molecular mass of the r*Ts*14-3-3 (with the histidine tag) was approximately 30 kDa, which corresponded well to the predicted size of the gene product. The r*Ts*14-3-3 was easily purified with Ni-affinity chromatography. The purified r*Ts*14-3-3 was able to be strongly recognized by the same *T. spiralis*-infected swine and mouse sera used for identifying the immunogenic antigens by immunoproteomic approach, but not by normal swine and mice sera (Figure 4B). The r*Ts*14-3-3 was recognized not only by the early infected swine sera (7 dpi) but also by the sera from swine infected with *T. spiralis* for up to 28 days (Figure 4C). The expression of native *Ts*14-3-3 was detected in both stages of *T. spiralis* by using mouse antisera against r*Ts*14-3-3 (Figure 4D).

## Discussion

In recent years, trichinellosis has become an emerging and re-emerging parasitic diseases and the severity of trichinellosis in human ranges from subclinical to fatal [24]. Early diagnosis of the infection is critical for the timely and effective treatment of trichinellosis because anthelmintic drugs are much more effective against adult worms in the intestine than to the encapsulated larvae in the muscle [9,12]. Therefore, it is important to identify antigens recognized by the host immune system during the early infection stage. These immunodominant antigens can be developed as biomarkers for the early diagnosis of trichinellosis or even as vaccine candidates to better control this food-borne zoonotic disease.

Although a number of *Trichinella* antigens, such as TSL-1, serine protease and histidine-tailed metalloprotein, have been identified as immunodominant antigens recognized by the host immune system during natural infection [8,25,26], these antigens do not meet the requirements for the early diagnosis of infection. Some somatic antigens from adult *T. spiralis* worms were recognized by *Trichinella* early or late infection sera from pigs by immunoscreening the adult worm cDNA library [27]. Therefore, it is possible to identify and develop these immunodominant antigens from adult worm as immunological reagents for the early diagnosis of *Trichinella* infection because the adult stage only exists during the early stage of infection.

In this study, a novel immunoproteomics approach was used to identify potential immunogens for the early diagnosis of trichinellosis or to develop an effective vaccine. The soluble extracts from *T. spiralis* adult worms were separated by 2-DE and detected with early infection sera from swine or mice infected with *T. spiralis* for only 7 days. During this period of infection, *Trichinella* infective larvae develop only into adult worms in the intestine of the infected host; therefore, the antigens from the adult worms that elicit an early immune response in infected animals may be used as potential target antigens for early diagnosis and/or vaccines against trichinellosis.

More than 60 *T. spiralis* adult worm proteins were recognized by the pooled early infection sera from infected pigs and mice, and 55 of these proteins were identified by MALDI-TOF/TOF-MS. The MW of the identified antigens ranged from 12.3 kDa to 93.7 kDa, and the pI ranged from pH 5.0 and 9.5. The fifty-five identified antigens represent 40 different proteins. Several antigens had more than one spot with different pIs, such as intermediate filament protein, actin-5c, 14-3-3, inorganic pyrophosphatase, putative thioredoxin, and serine protease inhibitor. It is possible that these antigens have more than one isoform with different post-translation modifications or binding to different co-factors that change their pI or molecular weight. Remarkably, these proteins are involved in a wide range of biological functions, such as biological regulation, cellular process, metabolic process, and localization.

Among the potential immunogens in present study, several structural proteins, including cytoskeleton and muscle proteins, such as actin-5c was also identified. Studies indicated that actin is related to the invasion of worm into the intestinal epithelial cells, indicating this protein may be involved in the early survival of the parasites within the host [28]. Mice infected with *Echinostoma caproni*, an intestinal trematode, produce early immune responses to actin, suggesting that this protein is exposed early to the host and may be of importance in the establishment of parasitism [29].

Heat shock proteins (HSPs) are traditionally considered a highly immunogenic and conserved family of proteins. These proteins were identified as potential vaccine candidate antigens and may also function as immunological modulators of immune responses [30]. Of these, HSP70 is a major target of immune responses of the host to infections by helminths and protozoan parasites [31,32]. Previous studies indicated that SmHSP70 elicited an early humoral immune response and may be a good target for the immunodiagnosis of schistosomiasis [33]. HSP70 in *Echinostoma caproni* also induced a strong early immune response in mice [29]. Moreover, our previous study indicated that *Ts-*Hsp70 from adult *T. spiralis* is highly immunogenic and may be a good vaccine candidate [34,35]. In this study, *Ts*-HSP70 was recognized by early infection sera, indicating that this protein has potential for the early diagnosis of trichinellosis.

Another interesting protein recognized by early infected sera in this study is cysteine protease. This protease is a key molecule in host-parasite interactions, which has gained significant attention as a target for chemotherapy or immunoprophylaxis [36]. Vaccination with cysteine protease coding DNAs induced significant protection against several helminth and protozoan infections by reducing the parasite burden or fecal egg count and reducing egg viability [37]. In addition, many cysteine proteases can be used for the immunodiagnosis of parasitic diseases with good sensitivity and specificity [38,39]. Recognition of the cysteine protease by early *T. spiralis* infection sera in this study suggests that *T. spiralis* cysteine protease is not only a good target for vaccine but also a promising target for immunodiagnostic antigen.

Of the immunogens identified in the present study, the 14-3-3 protein is of particular interest. There were two protein spots that matched the 14-3-3 protein of *T. spirali*, which indicates that the 14-3-3 protein was a strong immunogenic antigen during the early infection. The 14-3-3 proteins, a series of abundant acidic proteins which belong to a family of conserved regulatory molecules, play key roles in several eukaryotic biochemical processes, such as signal transduction, transport, regulation, cell differentiation and cell survival, and have been identified in several helminth parasites [40,41]. Studies indicated that this protein could be used for routine immunodiagnosis and serological epidemiological surveys as well for helminthic diseases with high sensitivity and specificity [42,43]. In addition, for parasites, such as *S. mansoni* and *E. granulosus*, the 14-3-3 proteins can induce humoral and cellular immune responses and have been tested as potential vaccine targets [44,45]. Therefore, it intrigues our interest to evaluate *Ts*14-3-3 identified in this study as a reagent for the early serologic diagnosis for trichinellosis or even for the target of vaccine against *Trichnelle* infection. Primary expression assay demonstrated that *Ts*14-3-3 is feasible to be expressed as soluble recombinant protein in *E. coli* and the purified recombinant *Ts*14-3-3 from induced bacterial lysates was able to be recognized not only by the early *T. spiralis*-infected sera from swine and mice (7 dpi), but also by sera of swine infected for up to 28 days. In addition, this antigen exists in both adult and muscle larvae stages, indicating this *Ts*14-3-3 can be used not only for early diagnosis but also for detecting whole period of infection. These results further confirm that *Ts*14-3-3 is an immunodominant antigen recognized by *T. spiralis* infected sera and could be a good target for early immunodiagnosis of trichinellosis. The sensitivity and specificity of recombinant *Ts*14-3-3 as an antigen for serological diagnosis of *Trichinella* infection is under investigation by testing the defined sera from patients with trichinellosis compared with normal sera and sera from patients with other helminthiases.

In this study, we identified candidate antigens for early immunodiagnosis and vaccine development against trichinellosis using an immunoproteomics approach. The proteins identified in this study, specifically *Ts*14-3-3 protein, cysteine protease and HSP70, may play critical roles in the maintenance of the host-parasite relationship during the early stage of *Trichinella* infection, therefore are good targets for vaccine and early immunodiagnosis as well. Except for *Ts*14-3-3 that has been successfully expressed in *E. coli* as soluble recombinant protein that can be recognized by *Trichinella*-infected swine and mouse sera, other identified antigens are also being evaluated for their potential as immunodiagnostic or vaccine reagents using *in vitro* expression of the recombinant proteins, immunological test for defined infection sera and challenge study with *T. spiralis* infection animal model.

## Conclusions

In this study, 2-DE and Western blot were used to characterize the diagnostic antigens or vaccine candidates from *T. spiralis* adult worm. A total of 64 proteins from the adult worm were recognized by early infection sera from pigs and mice infected with *T. spiralis* for 7 days. The proteins identified in this study, especially 14-3-3 protein, possess significant potential as early diagnostic reagents or vaccine candidates.
